# Larval species diversity, seasonal occurrence and larval habitat preference of mosquitoes transmitting Rift Valley fever and malaria in Baringo County, Kenya

**DOI:** 10.1186/s13071-019-3557-x

**Published:** 2019-06-11

**Authors:** Isabella M. Ondiba, Florence A. Oyieke, Duncan K. Athinya, Isaac K. Nyamongo, Benson B. A. Estambale

**Affiliations:** 1 University of Nairobi, Nairobi, Kenya; 2 Jaramogi Oginga Odinga University of Science and Technology, Bondo, Kenya

**Keywords:** Baringo, Species diversity, Larval habitats, Malaria, RVF, Season

## Abstract

**Background:**

Baseline information that is essential for determining the areas to target with larval control includes estimates of vector diversity and larval habitat preferences. Due to a lack of such information in Baringo County, Kenya, this study assessed species diversity and larval habitat preference of potential mosquito vectors of Rift Valley fever (RVF) and malaria.

**Methods:**

Mosquito larvae were sampled from nine types of larval habitats and were identified morphologically. Species diversity was estimated by the Shannon’s diversity index while larval habitat preference by RVF and malaria vectors was determined by ANOVA.

**Results:**

A total of 7724 immature mosquitoes comprising 17 species belonging to four genera, namely *Anopheles*, *Culex*, *Aedes* and *Mansonia*, were identified. Among the 17 species, three *Anopheles* species are responsible for malaria transmission: *An. gambiae* (*s.l.*), *An. funestus* (*s.l.*) and *An. pharoensis*. Rift Valley fever vectors included *Mansonia* spp. and *Culex* spp. The highest Shannon's diversity index was observed during the cold dry season (*H* = 2.487) and in the highland zone (*H* = 2.539) while the lowest diversity was recorded during the long rain season (*H* = 2.354) and in the riverine zone (*H* = 2.085). Ditches had the highest mean number of *Anopheles* larvae (16.6 larvae per sample) followed by swamp (12.4) and seasonal riverbed (10.7). Water pit and water pan had low mean numbers of *Anopheles* larvae (1.4 and 1.8, respectively) but relatively high mean numbers of culicines (16.9 and 13.7, respectively). Concrete tank was the least sampled type of habitat but had highest mean number of culicine larvae (333.7 l) followed distantly by water spring (38.9) and swamp (23.5). Overall, larval habitats were significantly different in terms of larval density (*F*_(8,334)_ = 2.090, *P* = 0.036).

**Conclusions:**

To our knowledge, the present study reports culicine larval species diversity in Baringo for the first time and the most preferred habitats were concrete tanks, water springs and swamps. Habitats preferred by *Anopheles* were mainly riverbed pools, ditches and swamps. Environmental management targeting the habitats most preferred by potential vectors can be part of integrated vector control in Baringo, especially during dry seasons.

## Background

More than 80% of the world’s population is at risk of one or more vector-borne diseases [1]. Mosquitoes are responsible for most vector-borne disease transmission and Africa bears a large burden [2]. Out of the 3000 known species of mosquitoes, about 100 are vectors of human diseases [3]. The common diseases transmitted by mosquito vectors include Rift Valley fever (RVF) and malaria. Rift Valley fever is a zoonotic disease transmitted by a *Phlebovirus* of the family Bunyaviradae [4]. Eleven epizootics have occurred in Kenya between 1951 and 2007 with an average inter-epizootic period of 3.6 years [5]. During the last RVF outbreak in Kenya in 2006/2007, the highest proportions of cases (31%) were from Garissa in the northeastern region followed by Baringo (24%) in the Rift Valley region. This was the first time RVF transmission was reported in Baringo [6].

Malaria is another vector-borne disease transmitted by mosquitoes. Global estimates of malaria cases and mortality were 212 million and 429,000, respectively, in 2015 with 90% of the cases occurring in sub-Saharan Africa [7]. This calls for the up-scaling of control strategies and inclusion of more innovative ways to supplement existing interventions. Malaria is a prevalent disease in Baringo, accounting for 11.8% of outpatients in health facilities [8]. The burden of malaria in Baringo is higher in the low-lying areas where transmission occurs throughout the year [9] but cases of malaria also occur in the highlands [10]. Malaria fatalities increase during outbreaks in areas where populations are immunologically vulnerable [11]. Such cases of explosive malaria were witnessed in October 2017 in Baringo East, a midland area that is rarely affected by malaria.

The current vector control strategies in Kenya, such as LLINs and IRS which target indoor resting mosquitoes, are insufficient. These strategies may not protect against outdoor resting mosquito vectors such as the culicine species that transmit RVF and secondary malaria vectors like *An. pharoensis* and *An. coustani*. Larval source management (LSM) should, therefore, be an additional strategy to supplement the existing interventions as part of an integrated vector management (IVM) policy [12]. This is possible because larval habitats in Baringo are mainly permanent artificial water bodies which are few in number, accessible and easily identifiable [13]. Information on the diversity and distribution of endemic vector species is essential and requires knowledge on the identity of mosquito species present in each locality for effective implementation of vector management [14–16]. The present study determined species diversity, seasonal occurrence and larval habitat preference by RVF and malaria vectors in Baringo.

Factors that affect mosquito species diversity include season, elevation and type of aquatic habitat [17]. Mosquito diversity parameters like species richness and abundance can be compared between different ecological zones and seasons. Areas with more diverse larval habitats are likely to have higher mosquito species diversity than areas with few larval sites [18]. Seasonal changes can affect larval habitat availability and productivity and thereby impact on species diversity. It is important to sample the same area continuously to cover different seasonal climatic conditions [17]. In sub-Saharan Africa, a significant decrease in the spatial distribution of larval habitats during the dry season [19] could affect species diversity. A survey of the Mara river basin found the highest number of mosquito larvae during the dry season at the dry stream beds compared to other habitat types [20].

Several studies have shown that different mosquito species prefer different larval habitats. *Anopheles gambiae* (*s.l.*), the principal vector of malaria, prefers slightly turbid, shallow, sunlit and transient water pools without aquatic plants [21, 22]. *Anopheles pharoensis*, a secondary vector of malaria, breeds in habitats with floating vegetation and with relatively shady conditions [22]. A recent study found no *Anopheles* larvae in an abandoned fishpond [23], a confirmation that *Anopheles* species do not prefer deep water bodies [24], probably due to lack of siphon used for breathing under water. However, it was not possible to identify clear characteristics of larval habitats for *Anopheles* species larvae in Tanzania [25]. Culicine mosquitoes have been shown to exploit a wide range of aquatic habitats with slight differences among individual species [18, 26]. All potential larval habitats can have one or more larval species of the genus *Culex* [18, 27]. *Mansonia* mosquitoes prefer habitats with aquatic plants such as *Pistia* spp. and polluted water [28–30]. The *Mansonioides* prefer habitats with well-developed macrophytes which provide mechanical support and favorable conditions for oviposition. *Aedes* spp. exploit a wide range of larval habitats with different temporal characteristics [31], probably due to adaptation to broad environmental components of physico-chemical factors. Specifically, *Aedes aegypti* mosquitoes prefer larval habitats of artificial water containers [32, 33].

Kenya is divided into four epidemiological regions based on malaria. A lot of research on larval vector surveys has been conducted in the endemic regions of the lake Victoria basin (western Kenya), central Kenya and coastal regions [16, 34–37] but few larval studies have been conducted in the semi-arid, seasonal transmission areas such as Baringo [13, 38]. Although some research has been performed in Baringo on mosquito vectors, there is limited information on species diversity and larval habitat preference. Furthermore, such previous studies did not cover the entire county because they were limited to areas around Lake Baringo and Lake 94. The aim of the present study was to investigate larval species diversity, seasonal occurrence and habitat preference of RVF and malaria vectors in a wider area of Baringo categorized into four ecological zones. Knowledge on seasonal mosquito species diversity and larval habitat preferences of the vectors will allow public health officials to more accurately carry out targeted larval source management in Baringo.

## Methods

### Description of the study area

**Fig. 1:**
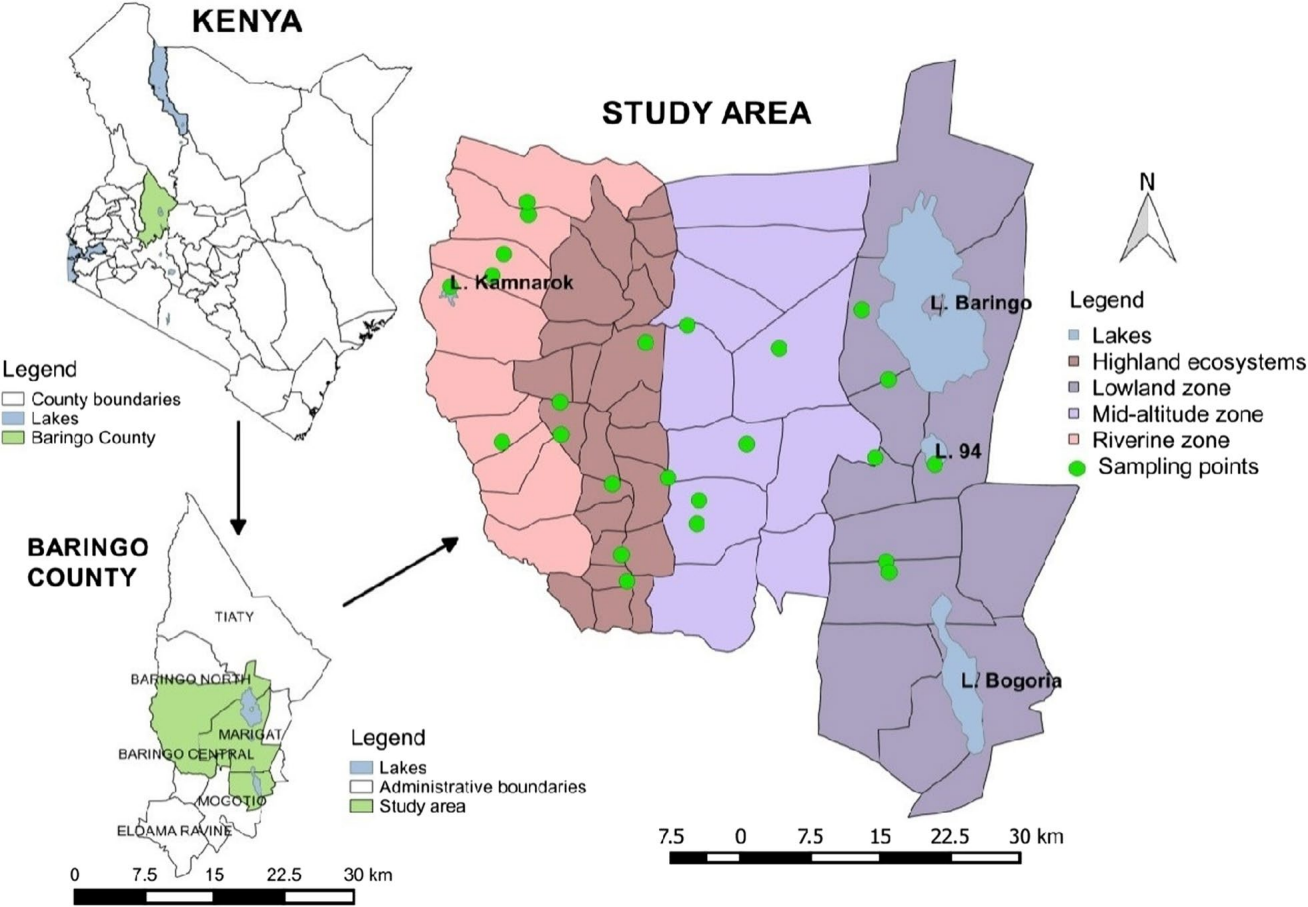
Map of study area in Baringo County

The study was conducted in Baringo County, Kenya. The area surveyed was between 0°32′28″–0°43′23″N, 35°36′7″–36°16′37″E at an altitude ranging between 870 and 2499 m above sea level (Fig. 1). The study area was divided into four ecological zones: lowland, midland, highland and riverine. Baringo is characterized by four lakes, Lake Baringo, Lake Bogoria, Lake 94 and Lake Kamnarok. The seasonal rivers in Baringo are often characterized by pockets of small pools of water along the riverbed, which provide suitable larval habitats for mosquitoes. Dams also exist, which form focal points where humans and livestock aggregate to access water, especially during the dry season.

Baringo County has two distinct weather patterns, namely dry and wet seasons. The dry season consist of months with distinctly low temperatures (June to August) and those with high temperatures (December to February). Mean monthly temperatures in the highlands range from 25 °C during the cold months to 30 °C during the hot months, while in the lowlands it ranges between 30 and 35 °C during cold and hot months, respectively. Baringo County experiences two rainy seasons: long rains (March to May) and short rains (September to November). The County experiences extreme spatial fluctuations in seasonal rainfall. It receives between 1000–1500 mm of rainfall annually in the highlands and 500–600 mm in the lowlands [39]. Monthly temperature and rainfall for Baringo during the study period (2014–2016) were obtained from IRI and CHIRPS [40].

### Larval habitats, sampling and identification of mosquitoes

**Fig. 2:**
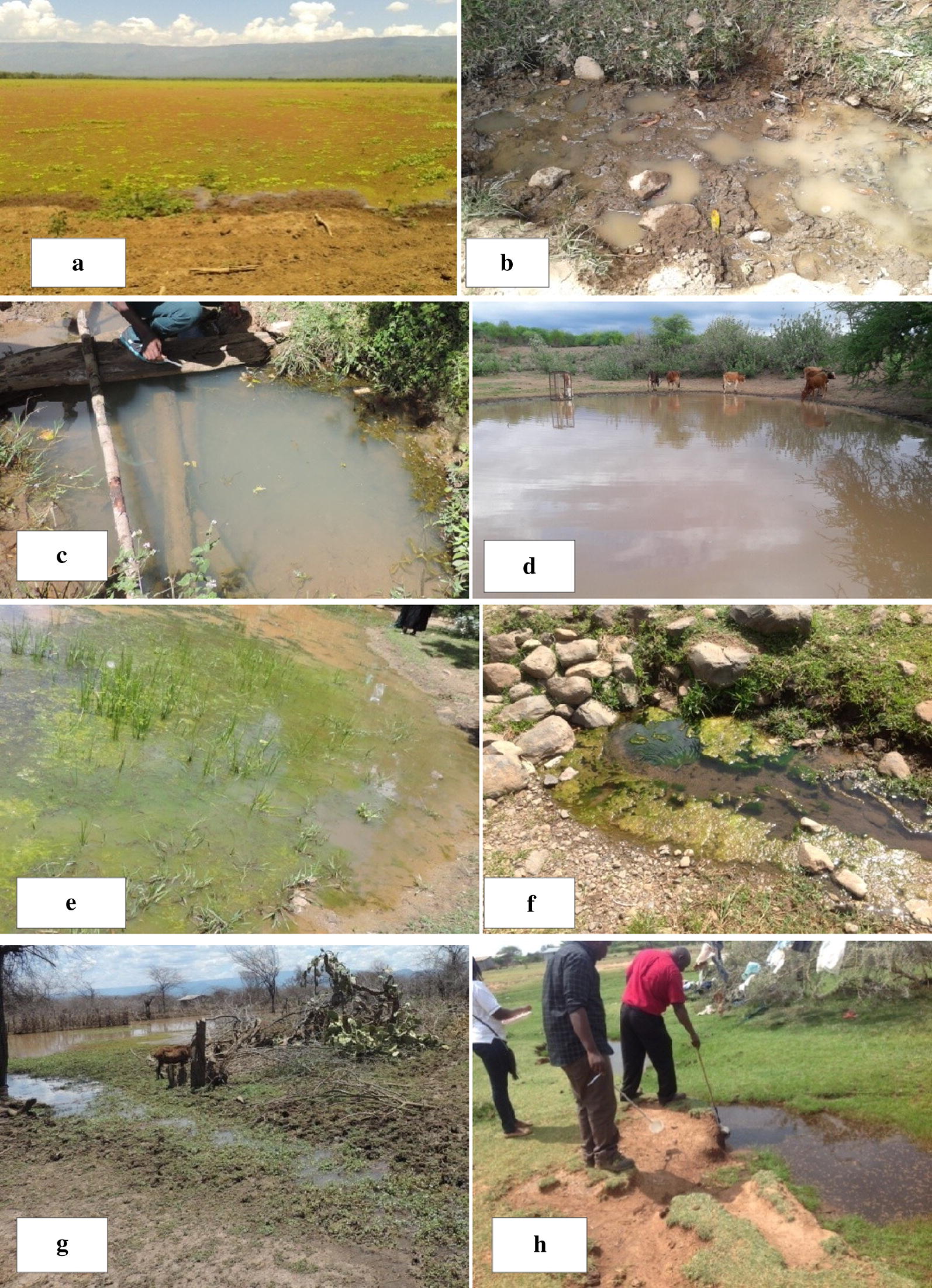
Larval habitats that were sampled regularly in Baringo County. **a** Lake Kamnarok covered with small floating plants. **b** Water pit with hoof prints. **c** Water spring. **d** Water pan without vegetation. **e** Water pan with algae and grass. **f** Riverbed pool. **g** Lake Baringo at Salabani. **h** Ditch

Twenty-four sites (six from each ecological zone) with potential mosquito larval habitats were identified and mapped with geo-positioning equipment (GPS) during a preliminary survey. Larval habitats were selected to represent the diverse larval habitats (Fig. 2) in the heterogeneous topography of Baringo ecological zones. The same larval habitats were sampled longitudinally once every month between June 2014 and June 2016. Larvae collected monthly were consolidated into seasons then analyzed to assess seasonal fluctuations in species diversity and density per dip. Ten to twenty dips were taken depending on size of habitat using the 350 ml standard dipper. Different points along the edge of large habitats such as lake shore were sampled hence more dips than those taken from smaller habitats such as water springs. However, the main objective was to find out the species found in different habitat types without considering the habitat size. The larvae were transferred into a sample container using a wide-bore pipette. Larvae were morphologically identified under a dissecting microscope to the lowest possible taxonomic unit [41, 42].

### Statistical analyses

#### Analysis of species diversity

Average monthly temperature and rainfall during the sampling period were used to represent the seasonal climatic conditions. Information on species diversity in Baringo County for each ecological zone and season were estimated using Shannonʼs diversity index. Shannonʼs index was selected because it combines species richness and abundance and is also sensitive to rare and abundant species [43]. Pairwise comparisons of larval species diversity between ecological zones and seasons were made using the Shannonʼs diversity t-test as proposed by Hutcheson [44] based on the following equation:

$$t = \frac{{H^{\prime}_{1} - H^{\prime}_{2} }}{{S_{{H^{\prime}_{1} - H^{\prime}_{2} }} }}$$

where $S_{{H^{\prime}_{1} - H^{\prime}_{2} }} = \sqrt {S_{{H^{\prime}_{1} }}^{2} + S_{{H^{\prime}_{2} }}^{2} }$with each variance of $H^{\prime}$ estimated by

$$S_{{H^{\prime}}}^{2} = \frac{{\sum {fi\,{ \log }^{2} \,fi - \left( {\sum {fi\,\log \,fi} } \right)^{2} } /n}}{{n^{2} }}$$

where $H^{\prime}$ is the Shannonʼs diversity index for each of the two samples, *S* is species richness (total number of species), *S*^2^ is the variance of each sample ($H^{\prime}_{1}$ and $H^{\prime}_{2}$), *n* is the total abundance (number of individuals) and *fi* is the proportion each species makes towards total.

Simpsonʼs index was included to measure species evenness. Berger-Parkerʼs index was also used to indicate the proportion of the most abundant species in each ecological zone and climatic season. Species rarefaction curves were used to estimate sampling sufficiency and expected occurrence of species for smaller groups. Cumulative species abundance (ln S), Shannonʼs index (*H*) and log evenness (ln E) (SHE) profiles were used to estimate ecological heterogeneity.

#### Analysis of larval habitat preference

Larval density per dip was determined by dividing total number of larvae by number of dips to get the mean. The mean was then standardized by multiplying by highest number of dips since different numbers of dips were taken (10–20) based on size of larval habitat. A test of the data for normality using the Shapiro-Wilk test showed a non-normal distribution. Thus, data were log-transformed [log_10_ (n+1) because the data had many zeroes] to reduce skewness and improve normality. After the transformation, data were re-tested and found to have a normal distribution. One-way analysis of variance (ANOVA) was then used to compare mean larvae in each habitat so as to determine habitat preference by malaria and RVF vectors. When significant differences were observed in ANOVA, Tukeyʼs *post-hoc* test was used for pairwise comparisons of the means [18].

## Results

### Larval species diversity in ecological zones and seasons in Baringo

A total of 7724 immature mosquitoes comprising of 17 species belonging to 4 genera (*Anopheles*, *Culex*, *Aedes* and *Mansonia*) were identified from various larval habitats in the four ecological zones. The 17 species included five *Anopheles* species, three *Aedes* species, eight *Culex* species and one *Mansonia* species. The *Mansonia* species were collected at pupa stage and left to emerge before identification was done. Among the 17 species identified, three *Anopheles* species were malaria vectors: *An. gambiae* (*s.l.*) (8.1%), *An. funestus* (*s.l.*) (0.1%) and *An. pharoensis* (15.4%).

The Shannonʼs diversity index (Table 1) was highest in the highland zone (*H* = 2.539) followed by the lowland zone (*H* = 2.536), midland zone (*H* = 2.327) and riverine zone (*H* = 2.085). *Anopheles pharoensis* was the dominant species in the riverine zone and accounted for 18.9% of the total number of larvae collected in the riverine zone (Berger-Parkerʼs index = 0.189). A pairwise comparison of species diversity between zones by Shannon diversity t-test showed that only highland and riverine zones were significantly different in species diversity (*t*_(15.876)_ = − 2.534, *P* = 0.049). The Simpsonʼs index was highest (1-D = 0.915) in the highland zone, indicating a high level of species evenness, and lowest (1-D = 0.860) in the riverine zone, an indication that species were not evenly represented.

**Table 1:** Species diversity of larval mosquitoes across four ecological zones in Baringo County

Species	Highland	Lowland	Midland	Riverine
*An. gambiae* (*s.l.*)	37	228	275	76
*An. pharoensis*	37	595	331	211
*An. coustani*	7	45	31	1
*An. funestus* (*s.l.*)	0	6	0	0
*Cx. pipiens*	538	116	216	19
*Cx. quinquefasciatus*	823	865	289	190
*Cx. annulioris*	108	125	658	78
*Cx. poicilipes*	128	34	60	10
*Cx. tigripes*	95	34	26	0
*Cx. dutoni*	131	19	9	2
*Ae. taylori*	12	0	0	0
*Ae. aegypti*	18	1083	8	0
*Ae. africanus*	7	8	0	0
*Mansonia* spp. pupae	23	3	5	1
*Cx. univittatus*	0	6	0	0
*Cx. vansomereni*	1	0	0	0
*An. rufipes*	0	0	0	2
No. of species	14	14	11	10
Simpsonʼs index (1-D)	0.9154	0.9143	0.897	0.8597
Shannonʼs index (*H*)	2.539	2.536	2.327	2.085
Berger-Parkerʼs index	0.1271	0.1235	0.1284	0.1893

**Fig. 3:**
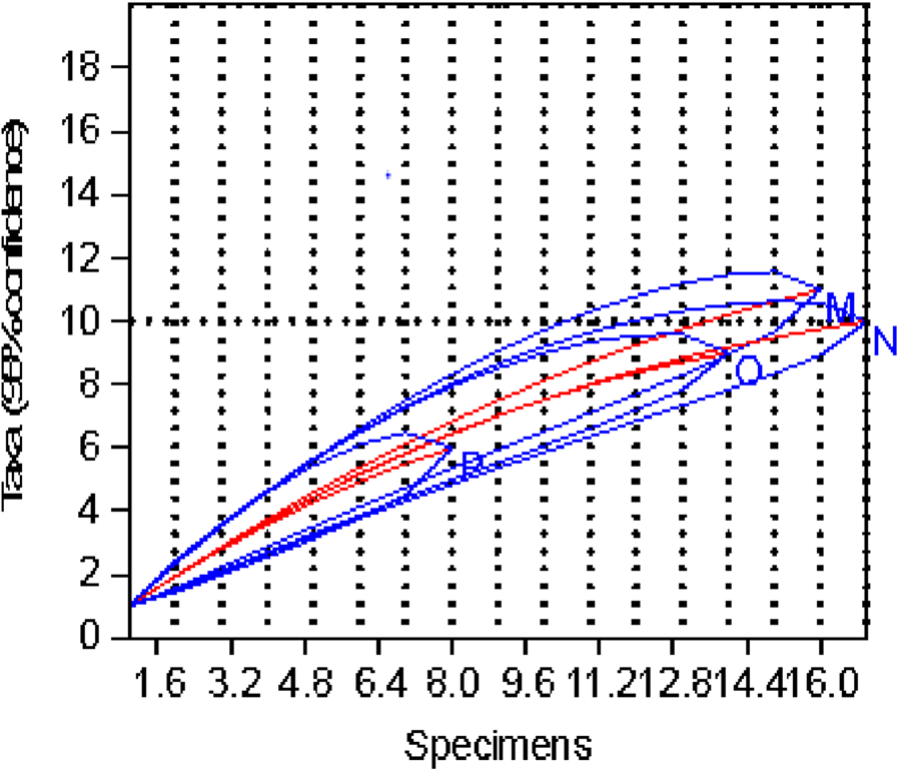
Rarefaction curves of the number of species against number of samples for ecological zones. *Abbreviations*: M, highland; N, lowland; O, midland; P, riverine

The rarefaction curve for the riverine zone (P in Fig. 3) showed that the common six species were obtained after 8 samples while for the highland zone (N in Fig. 3), the common ten species were obtained after 17 samples. Similarly, no new species were collected from the midland and lowland zones after 14 and 16 samples, respectively.

**Fig. 4:**
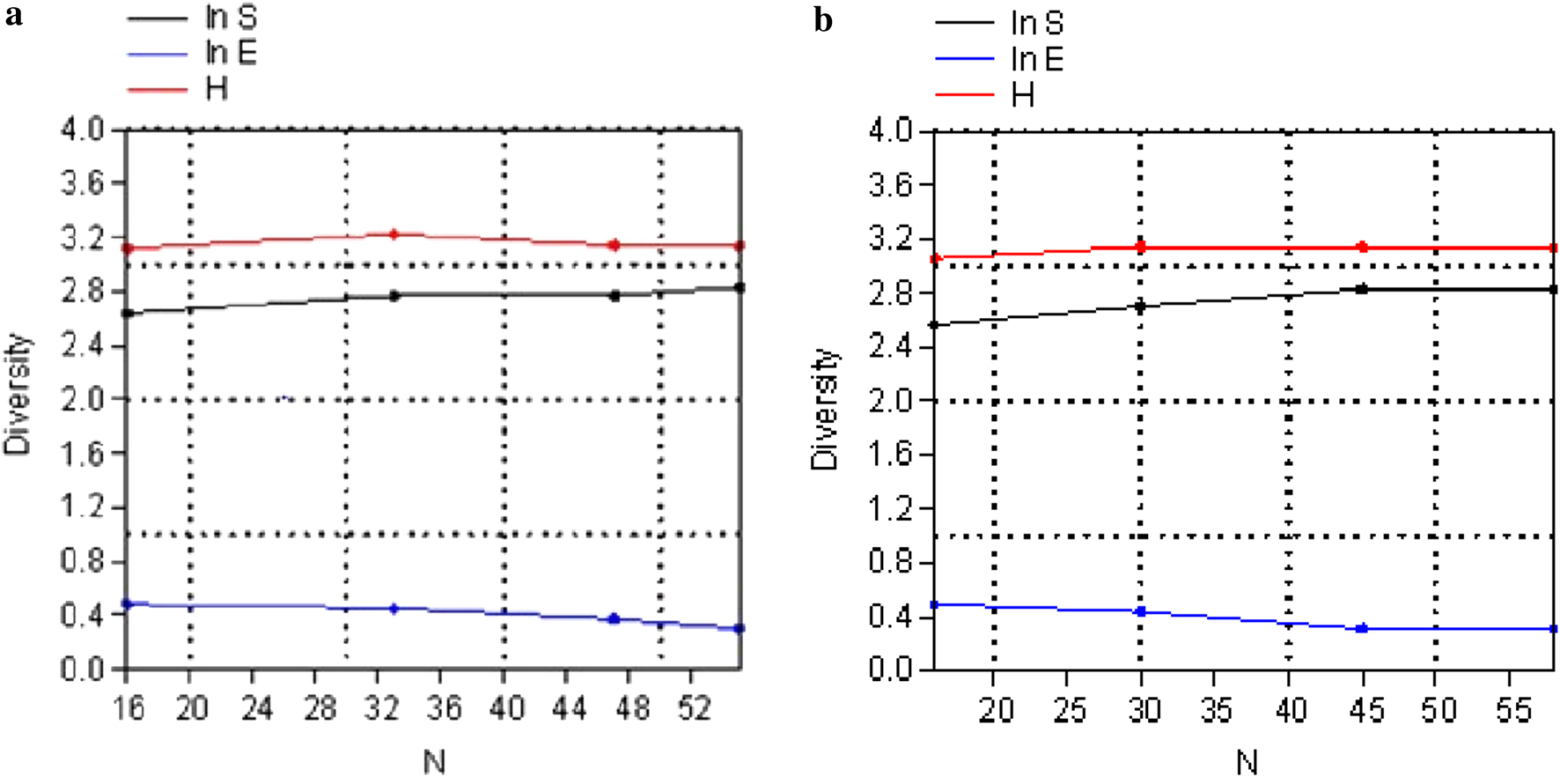
**a** SHE profiles for zones. **b** SHE profiles for seasons. *Key*: ln S, number of species (taxa); ln E, evenness; H, Shannonʼs index

The combined plots of cumulative species abundance (ln S), Shannonʼs index (*H*) and log evenness (ln E) (SHE) profiles showed that the four ecological zones were not obviously heterogeneous as the lines representing each measurement did not change much in direction (Fig. 4a). Similarly, species abundance, diversity and evenness were not different between seasons (Fig. 4b).

**Fig. 5:**
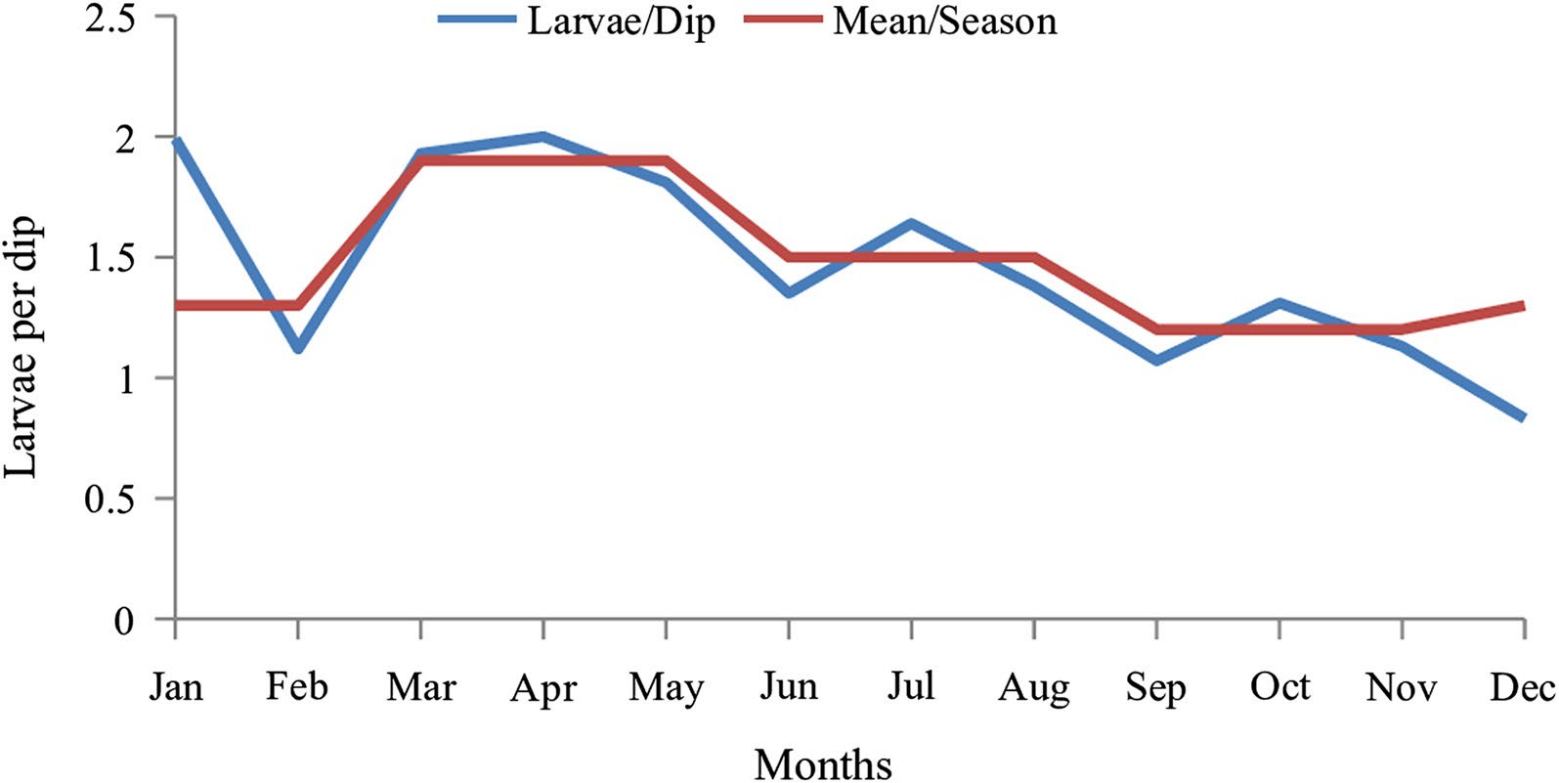
Monthly average larvae per dip and overall mean per season for all species

Monthly data analysis showed that the average number of larvae for all species combined per dip was high in April (2.0 larvae per dip), January (1.99), March (1.93) and May (1.81). A low density of larvae was observed in December and September, 0.83 and 1.07 larvae per dip, respectively. Overall, larval density was high in the long rain season followed by the cold dry season (Fig. 5).

**Fig. 6:**
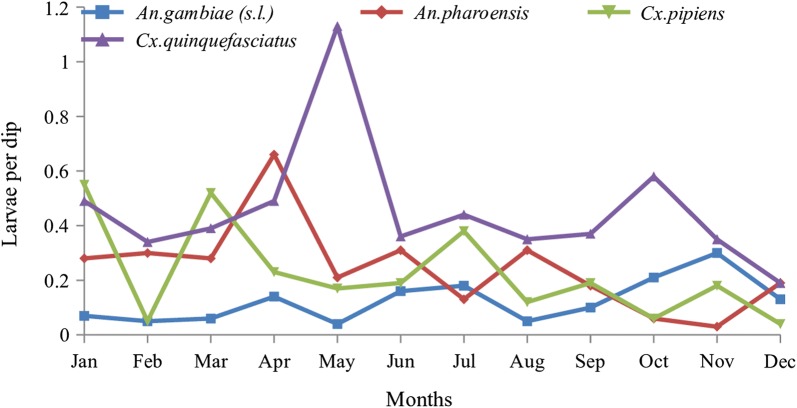
Monthly average larvae per dip for individual species

Although the trend for combined species showed high density during the long rain season (March to May), individual species showed different trends (Fig. 6). *Anopheles pharoensis* density was high in April during the long rain season and lowest in November (short rain season) when *An. gambiae* (*s.l.*) density was highest. On the other hand, *Culex quinquefasciatus* peaked in May during the long rain season but was lowest in December during the dry season.

*Anopheles gambiae* (*s.l.*) was notably low in abundance during the long rain season (*n* = 61) and the highest abundance was recorded during the short rain season (*n* = 263) while *An. pharoensis* was lowest in abundance (*n* = 127) during the short rain season. However, statistically there was no significant difference between seasons in *An. gambiae* (*s.l.*) abundance (*F*_(3,406)_ = 2.115, *P* = 0.098) but *An. pharoensis* was significantly different in abundance between seasons (*F*_(3,406)_ = 4.544, *P* = 0.004). On the other hand, *Mansonia* species which are the main vectors of RVF in Baringo constituted 0.42% while *Culex* species which are secondary vectors of RVF constituted 59.97%. The genus *Culex* was represented by 8 species dominated by *Cx. quinquefasciatus.*

The highest Shannonʼs diversity index (Table 2) of species was observed during the cold dry season (*H* = 2.487) whereas the lowest diversity was recorded during the long rain season (*H* = 2.354). *Culex quinquefasciatus* was the dominant species during three of the four seasons except the long rain season when *Aedes aegypti* was the dominant species constituting 42.9% of the total larvae (Berger-Parkerʼs index = 0.429). *Culex quinquefasciatus* constituted 40, 28.1 and 28.3% during the short rain, cold dry and dry season, respectively. However, these variations in abundance were not statistically different (*F*_(3,406)_ = 0.036, *P* = 0.991).

**Table 2:** Effect of season on species diversity of larval mosquitoes in Baringo County

Species	Cold dry season	Dry season	Long rain season	Short rain season
*An. gambiae* (*s.l.*)	179	113	61	263
*An. pharoensis*	346	375	326	127
*An. coustani*	77	3	0	4
*An. funestus* (*s.l.*)	6	0	0	0
*Cx. pipiens*	316	116	267	190
*Cx. quinquefasciatus*	533	497	568	569
*Cx. annulioris*	209	417	177	166
*Cx. poicilipes*	117	48	11	56
*Cx. tigripes*	62	43	29	21
*Cx. dutoni*	18	123	14	6
*Ae. taylori*	5	4	0	3
*Ae. aegypti*	5	4	1099	1
*Ae. africanus*	0	4	0	11
*Mansonia* spp. pupae	21	4	3	4
*Cx. univittatus*	0	0	6	0
*Cx. vansomereni*	0	1	0	0
*An. rufipes*	0	0	2	0
No. of species	13	14	12	13
Simpson’s index (1-D)	0.912	0.9069	0.8962	0.9008
Shannon’s index (*H*)	2.487	2.474	2.354	2.41
Berger-Parker’s index	0.1179	0.1276	0.1488	0.1442

Pairwise comparison between the four seasons by Shannonʼs diversity t-test showed that they were all significantly different from each other in species diversity. When the four seasons were merged into the two groups referred to as dry and wet seasons, still there was a significant difference (*t*_(7570.1)_ = 7.57, *P* < 0.0001) with the dry season having a higher species diversity than the wet season. The Simpsonʼs index was highest (1-D = 0.912) during the cold dry season indicating a high level of species evenness. The long rain season had the lowest Simpsonʼs index for species evenness (1-D = 0.896).

**Fig. 7:**
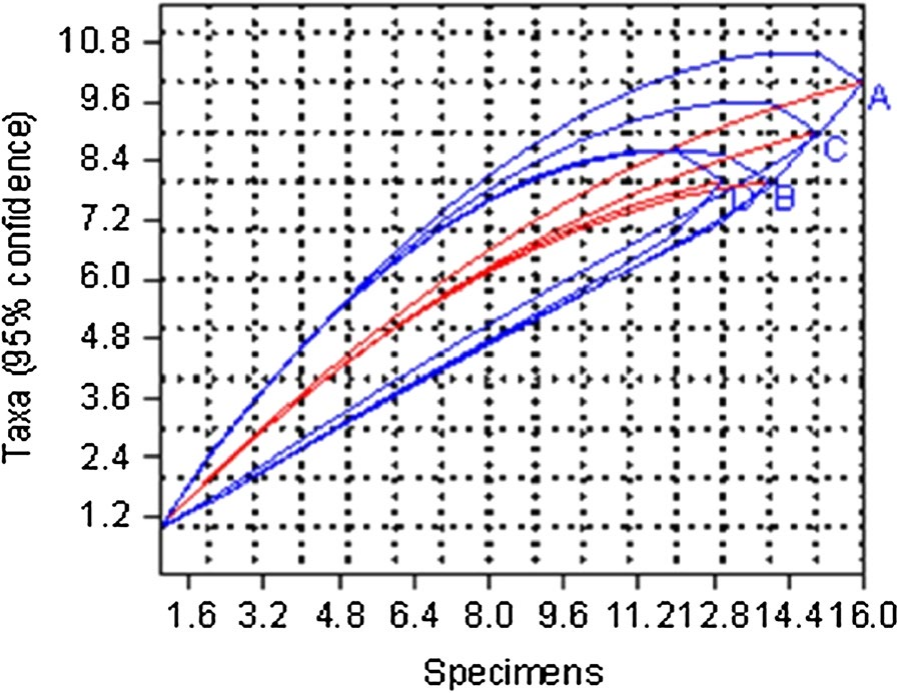
Rarefaction curves of the number of species against number of samples for seasons. *Abbreviations*: A, Cold dry season; B, Dry season, C, Long rain season, D, Short rain season

The rarefaction curve for the short rain season (D in Fig. 7) showed that the common eight species were obtained after 13 samples, while for cold dry season (A in Fig. 7), the common nine species were obtained after 16 samples. Similarly, no new species were collected during dry season and long rain season after 14 and 15 samples, respectively.

### Seasonality of larval habitats

**Fig. 8:**
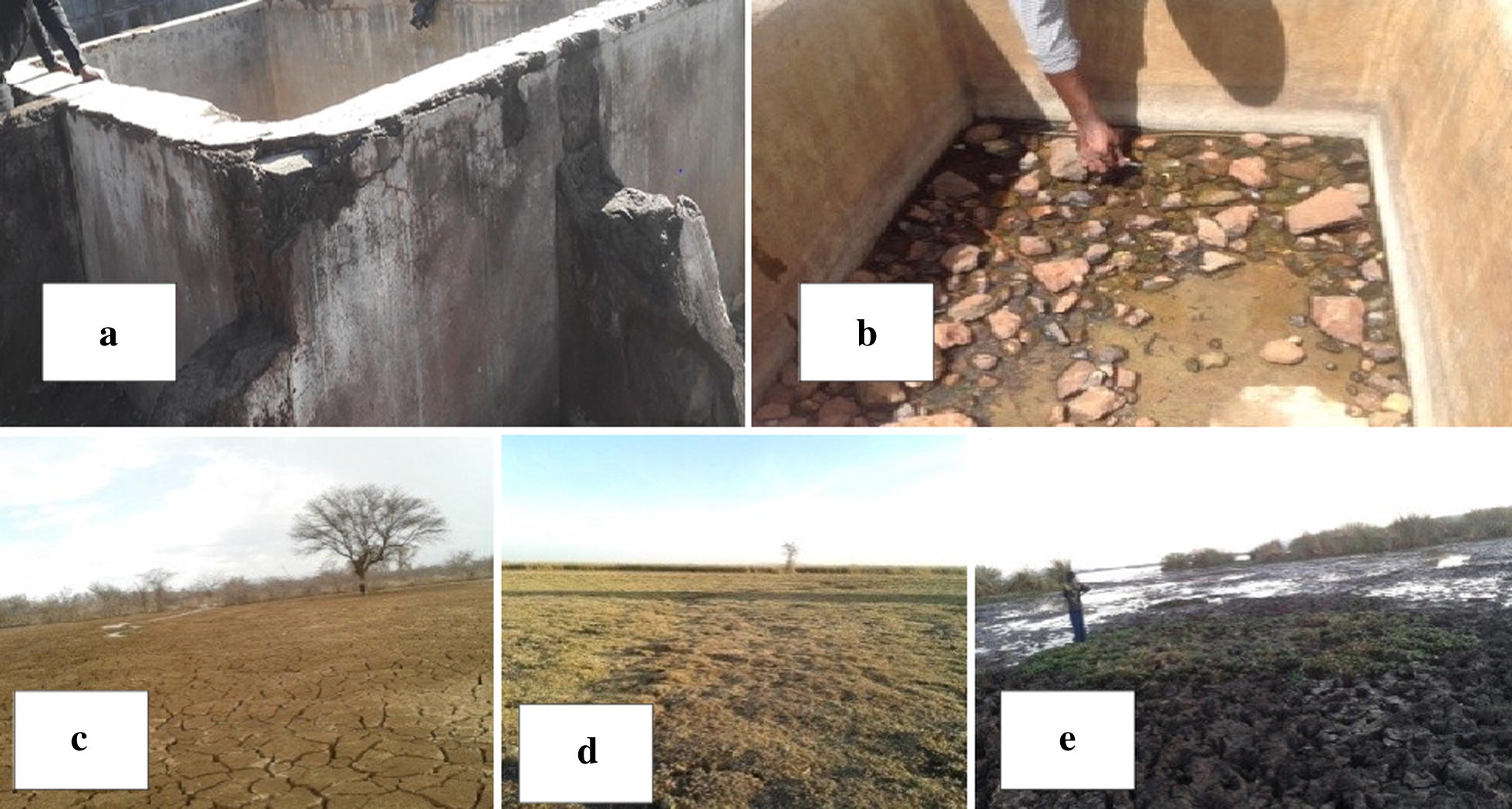
Larval sites that dried during the dry seasons in Baringo County. **a**, **b**, Concrete tank: **a** external view, **b** internal view during cold dry season. **c** Dried edge of Lake Baringo at Salabani. **d** Dried edge of Lake 94. **e** Wet area inside Lake 94

Nine categories of larval habitats were sampled, and the most commonly inhabited were lake margins, ditches, swamps, seasonal river beds, water pits, water pans and water springs. The least preferred habitats were the dam, which was consistently sampled, and concrete tanks, which were sampled only when they contained water (Fig. 8a, b). Lake margins receded during the dry season making sampling unfeasible. This happened at the Salabani sampling site on the shores of Lake Baringo and at the Sirata sampling site at the swampy Lake 94 (Fig. 8c–e). Water pans and some water pits also dried completely during the dry season. Swamps, ditches, water springs and riverbed pools persisted throughout the sampling period although the volume of water decreased in water springs and river-bed pools during the dry season.

A total of 29 larval habitats classified into 9 categories were sampled for several months when they contained water. Overall, 411 samples were taken cumulatively from all habitats during the study duration (2014–2016) and the most sampled habitat type was seasonal river bed at 5 sites totaling 81 samples (Table 3). This was closely followed by Lake Margin at 4 sites and water spring at 3 sites. The habitats that were least in number were dams and swamps, one each at two sites.

**Table 3:** Mean number of larval species in different habitat types

Habitat type	No. of habitats (%)	No. of samples	No. of anophelines collected ×20 dips	Mean per sample	No. of culicines collected ×20 dips	Mean per sample
Concrete tank	3 (10.3)	7	18	2.6	2336	333.7
Dam edge	2 (6.9)	25	164	6.6	333	13.3
Ditch	3 (10.3)	54	896	16.6	546	10.1
Lake margin	4 (13.8)	63	477	7.5	1099	17.4
River-bed	5 (17.4)	81	867	10.7	1444	17.8
Swamp	2 (6.9)	35	434	12.4	799	23.5
Water pan	3 (10.3)	27	48	1.8	370	13.7
Water pit	4 (13.8)	50	70	1.4	844	16.9
Water spring	3 (10.3)	69	271	3.9	2682	38.9
Total	29	411	3245		10,453	

### Larval habitat preference by culicines and *Anopheles* species

The ditch had the highest mean of *Anopheles* larvae (16.6 larvae per sample) followed by swamp (12.4 per sample) and seasonal river bed (10.7 per sample). The ditch was the only habitat which had higher mean of *Anopheles* larvae than culicine larvae. In the swamp, culicine larvae mean was almost double that of *Anopheles* larvae. Concrete tank was the least sampled type of habitat but had the highest mean number of culicine larvae (333.7 larvae per sample) with a low mean number of *Anopheles* larvae (2.6 larvae per sample). Water pit and water pan had the lowest mean number of *Anopheles* larvae, 1.4 and 1.8 larvae per sample, respectively (Table 3).

Overall, larval habitats were significantly different in terms of larval density (*F*_(8,334)_ = 2.090, *P* = 0.036). Multiple comparisons by *post-hoc* test showed that the concrete tank with a mean of 333.7 per sample was significantly different from dam edge, lake margin, river-bed, water pan and water pit. However, concrete tank was not statistically different from ditch, swamp and water spring which had relatively high mean number per sample. When concrete tank was excluded from the analysis because of its outstandingly high mean number, there was no significant difference between all habitats (*F*_(7,328)_ = 0.866, *P* = 0.534).

A separate analysis involving only *Anopheles* species showed that habitats were significantly different in terms of larval mean per sample (*F*_(8,401)_ = 9.595, *P* < 0.0001). Multiple comparisons by *post-hoc* test showed that the ditch which had the highest mean number of *Anopheles* larvae was significantly different (*P* < 0.05) from all other habitats except the swamp (*P* = 0.233), dam edge (*P* = 0.728) and concrete tank (*P* = 0.162). There was no significant difference between concrete tank and all other habitats in terms of *Anopheles* larval mean.

When analysis was performed for culicines only, there was also a significant difference between all sampled habitats (*F*_(8,401)_ = 4.903, *P* < 0.0001) and when concrete tank was excluded (*F*_(7,395)_ = 5.670, *P* < 0.0001). Concrete tank, dam edge and water pan were not significantly different from all other larval habitats in terms of the mean number of culicine larvae. On the other hand, water spring which had second highest mean number of culicine larvae was significantly different from ditch, lake margin and river-bed (*P* < 0.05). Although the swamp had a larval mean ranking 3rd highest, it was only different from ditch and lake margin.

### Occurrence of malaria and RVF vectors among the surveyed larval habitats

Out of the five *Anopheles* species identified, three are malaria vectors: *An. funestus* (*s.l.*), *An. gambiae* (*s.l.*) and *An. pharoensis*. *Anopheles funestus* (*s.l.*) was only collected from the ditch in the lowland zone while *An. gambiae* (*s.l.*) and *An. pharoensis* were collected from all types of larval habitats in all ecological zones. For *An. gambiae* (*s.l.*), riverbed pool had the highest mean number of larvae per sample (2.6) followed by ditch and swamp with means of 2.5 and 2.1 per sample, respectively. *Anopheles pharoensis* on the other hand had the highest mean of 6.9 larvae per sample in the ditch followed by swamp and riverbed pools with means of 4.0 and 3.5 per sample, respectively. Ditches, riverbed pools and swamp were the most three preferred larval habitats by malaria vectors followed by lake margin (Table 4).

**Table 4:** Malaria vector larval mean distribution in different larval habitats

Habitat type	No. of samples	Total no. of vectors	No. of *An. gambiae* (*s.l.*) (mean)	No. of *An. pharoensis* (mean)	No. of *An. funestus* (*s.l.*) (mean)
Concrete tank	7	8	1 (0.1)	7 (1.0)	0 (0)
Dam edge	25	65	5 (0.2)	60 (2.4)	0 (0)
Ditch	54	512	135 (2.5)	371 (6.9)	6 (0.1)
Lake margin	63	257	67 (1.1)	190 (3.0)	0 (0)
River-bed	81	491	207 (2.6)	284 (3.5)	0 (0)
Swamp	35	213	74 (2.1)	139 (4.0)	0 (0)
Water pan	27	24	13 (0.5)	11 (0.4)	0 (0)
Water pit	50	35	3 (0.1)	32 (0.6)	0 (0)
Water spring	69	144	65 (0.9)	79 (1.1)	0 (0)
Total		1749	570	1173	6

Further analysis to determine *An. gambiae* (*s.l.*) habitat preference showed a significant difference between habitats (*F*_(8,401)_ = 3.891, *P* < 0.0001). The *post-hoc* test showed that the water pit which had the least mean number of *An. gambiae* (*s.l.*) larvae per sample was significantly different from ditch (*P* = 0.001) and river-bed (*P* = 0.011) which had relatively high means. Water pan and water pit were the least preferred habitats by *An. gambiae* (*s.l.*). Analysis to determine habitat preference by *An. pharoensis* showed an overall significant difference between habitats (*F*_(8,401)_ = 5.949, *P* = 0.0001). The *post-hoc* test showed that the ditch which had the highest mean number of *An. pharoensis* was significantly different from river-bed and lake margin although they also had relatively high means (*P* < 0.005). There was no significant difference between the ditch and the swamp which had the second highest mean number of *An. pharoensis* larvae (*P* = 0.674).

*Mansonia* species, which are the only known vectors of RVF in Baringo, were collected from swamps, water pits, river-beds and water springs in small numbers. *Culex quinquefasciatus* and *Cx. pipiens*, which have only been implicated in RVF virus transmission, were collected from all habitats. Generally, *Cx. quinquefasciatus* was the most abundant larval species constituting 51.4% of potential arboviral larval species collected from all habitats. Of the three *Aedes* species collected, only *Ae. aegypti* and *Ae*. *africanus* are known vectors of yellow fever virus which is also an arbovirus belonging to the same group as RVF virus. *Aedes aegypti* and *Ae. africanus* were both collected from water pits, water pans and water springs in small numbers but a very large number of *Ae. Aegypti* was collected from concrete tanks. The concrete tank contributed 98.9% of *Ae. aegypti* larvae collected from all habitats and had a high mean of 155.9 larvae per sample indicating a high preference of water containers by this species (Table 5).

**Table 5:** Rift valley fever and other arboviral vector species distribution in all larval habitats

Habitat type	No. of samples	Total no. of vectors	No. of *Mansonia* spp. (mean)	No. of *Cx. quinquefasciatus* (mean)	No. of *Cx. pipiens* (mean)	No. of *Cx. univittatus* (mean)	No. of *Ae. aegypti* (mean)	No. of *Ae. africanus* (mean)
Concrete tank	7	1140	0 (0)	38 (5.4)	7 (1.0)	4 (0.6)	1091 (155.9)	0 (0.0)
Dam edge	25	125	0 (0)	93 (3.7)	32 (1.3)	0 (0)	0 (0)	0 (0)
Ditch	54	199	0 (0)	164 (3.0)	35 (0.6)	0 (0)	0 (0)	0 (0)
Lake margin	63	597	0 (0)	526 (8.3)	61 (1.0)	2 (0)	0 (0)	8 (0.1)
River-bed	81	638	5 (0.1)	325 (4.0)	308 (3.8)	0 (0)	0 (0)	0 (0)
Swamp	35	296	4 (0.1)	269 (7.7)	23 (0.6)	0 (0)	0 (0)	0 (0)
Water pan	27	201	0 (0)	198 (7.3)	1 (0.0)	0 (0)	0 (0)	2 (0.1)
Water pit	50	307	18 (0.4)	223 (4.5)	60 (1.2)	0 (0)	6 (0.1)	0 (0)
Water spring	69	715	5 (0.1)	331 (4.8)	362 (5.2)	0 (0)	12 (0.2)	5 (0.1)
Total		4212	32	2167	889	6	1103	15

Statistical analysis was performed only for *Cx. quinquefasciatus* because it was the most abundant arboviral vector. Although there was a significant difference in habitat preference by *Cx. quinquefasciatus* (*F*_(8,401)_ = 2.132, *P* = 0.032), it was only ditch and river bed that were different from swamp in terms of larval density. Swamp had the highest mean number of *Cx. quinquefasciatus* larvae while river-bed and ditch had low means (Table 5). All other larval habitats were not significantly different from each other in terms of larval mean for *Cx. quinquefasciatus*.

## Discussion

This study reveals a more complex larval species composition compared to previous studies in Baringo where only four anopheline species and one *Aedes* species had been identified [13, 33, 38]. *Culex* larvae have been collected from Baringo by other researchers but identification up to species level has not been undertaken [38]. The present study recorded larvae of 17 species, including *Anopheles* and culicines. The most favorable seasons for most mosquito species were cold dry and dry seasons as depicted by the high species diversity index. Therefore, the two seasons are appropriate for implementation of larval source management (LSM). Application of larvicides during these two seasons would be effective since there would be no wash off. Highland and lowland zones had high species diversity and should also be targeted for LSM.

The three *Aedes* species larvae collected in the present study, namely *Ae. aegypti*, *Ae. taylori* and *Ae. africanus*, are all vectors of arboviruses [45] but only *Ae. africanus* had previously been reported in high altitude woodlands in Baringo during the yellow fever outbreak of 1992–1993 [33]. The information on larval vector species can be instrumental for integrated control strategies in view of the fact that control of immature stages would be more appropriate since they are confined in small aquatic habitats where they cannot escape as opposed to adults which are highly mobile [13, 46].

*Mansonia* spp. are the main vectors of RVF in Baringo [47] but larval stages are not easy to find most likely because of their habit of attaching to aquatic plants [28, 29]. However, adult mosquitoes of *Mansonia* species have been collected from Baringo in the previous studies [47–50]. A few pupae of *Mansonia* species were collected during this study and were identified to genus level after emergence into adults. *Mansonia* species were found in habitats with vegetation. Removal of such vegetation can be an effective control method to prevent development of *Mansonia* [29] and hence reduce their population. Species of *Culex* were found in all habitats in the four ecological zones. This finding is consistent with previous studies in which one or more *Culex* species were found in all types of habitat [18, 27]. Whereas *Cx. quinquefasciatus* did not show a preference for any particular habitat across the four ecological zones, *Cx. univittatus* was only collected from the lake margin and concrete tank.

The *Aedes* species incriminated in the transmission of RVF virus (*Ae. mcintoshi* and *Ae. ochraceus*) develop and emerge from flood waters after unusually heavy and persistent rainfall [4]. However, these *Aedes* primary vectors of RVF have not been reported in Baringo, even during the previous active epizootics. *Aedes aegypti* specifically breed in containers but the present study focused more on sampling large and relatively permanent larval habitats. Nevertheless, sampling during the few months when rain was heavy yielded a large number of *Ae. aegypti* larvae from two concrete tanks in the lowland zone. The abundance of *Ae. aegypti* during the long rain season and its confinement to containers makes it easier to control at larval stage unlike adults which rest outdoors in diverse places [35]. A previous survey of domestic and peridomestic water receptacles in Baringo found no *Aedes* larvae except in one isolated cistern in Marigat town [33]. Similar results were reported from a study in Malaysia which revealed that indoor containers were more preferred larval habitats for *Ae. aegypti* [32]. *Aedes aegypti* is the primary vector of dengue, chikungunya and yellow fever viruses [33, 51–53]. Its presence in the lowlands is indicative of potential risks of spread of arboviruses in the event of an outbreak.

The presence of *An. gambiae* (*s.l.*), *An. funestus* (*s.l.*), *An. coustani* and *An. pharoensis*, previously reported in Baringo [13, 38] and confirmed in the present study, shows that they are the most predominant *Anopheles* species in the region. According to the study of Mala et al. [13], *An*. *arabiensis* is the most abundant species of *An. gambiae* complex in Baringo. *Anopheles gambiae* (*s.l.*) abundance was not significantly different among seasons. This is consistent with findings in western Kenya where no difference was found in *Anopheles* larval abundance between seasons [16]. Therefore, it is advisable to control malaria vector larvae in all seasons by targeting all habitats [38].

The present study shows that *Anopheles* species were distributed in all ecological zones (980–2200 m above sea level) except larvae of *An. funestus* (*s.l.*) which were found only in the lowland. This is consistent with studies conducted elsewhere in which it was found that malaria vectors are found in all levels of elevation [54]. The small and open, sunlit water pools preferred by *An. gambiae* (*s.l.*) [42, 55] were common in seasonal river beds in midland zone where this species was most abundant. This is similar to findings of studies conducted in Eritrea and Ethiopia where high larval productivity was recorded at stream bed pools [12]. This implies that riverbed pools could sustain malaria vectors responsible for transmission during the dry season in Baringo so they should be targeted for larval source management since they are easily identifiable. Findings from other studies link *An. gambiae* complex to artificial, environmentally-disturbed habitats and small shallow habitats without emergent vegetation [36, 37]. However, a study conducted in an urban environment in Tanzania demonstrated that it was not clear to define larval habitats for *An. gambiae* (*s.l.*) as high densities were found in polluted water [25]. In the present study, *An. gambiae* (*s.l.*) and *An. pharoensis* were found co-existing in the same larval habitats such as riverbed pools, ditches and lake margins. The riverbed pools and ditches were small in size and discrete hence can easily be treated to destroy larvae. On the other hand, *An. funestus* (*s.l.*) prefers deeper and more persistent habitats with vegetation [36]. The three species [*An. gambiae* (*s.l.*), *An. pharoensis* and *An. funestus* (*s.l.*)] appear to prefer different types of habitats which are all present in Baringo County, a factor that could be enhancing malaria transmission throughout the year.

Concrete tanks, water springs and swamps should be targeted for potential RVF vectors, while ditches and riverbed pools should be targeted for potential malaria vectors. Larval source management would reduce mosquito vector populations and supplement the current vector control strategies which target only indoor adult stages. Thus, larval source management is a feasible strategy than can be implemented in Baringo to control both indoor and outdoor resting mosquitoes such as *An. pharoensis* and culicine species.

## Limitations of the study

*Anopheles gambiae* (*s.l.*) larvae were not identified to the species level by molecular techniques and *Mansonia* species were not identified to the species level. This study focused on identifying larval sites, seasonal effects and species diversity and therefore sizes of larval habitats were not quantified. Future studies, aimed specifically at productivity of larval habitats, should take into account measurements of such sites and perform molecular identification of species complexes.

## Conclusions

The present study reports a higher diversity of culicine and *Anopheles* larvae than previous studies in Baringo. Occurrence of *Mansonia* species, *Aedes* species and several species of *Culex* indicates the potential for a rapid spread of arboviral diseases such as RVF and yellow fever which have been reported in Baringo in previous years. The most important larval habitats were riverbed pools, ditches, swamps and lake margins which should be targeted during larval control operations. The presence of malaria vectors in all seasons implies that transmission of malaria occurs throughout the year unlike previous assumptions that malaria transmission is seasonal in semi-arid areas. Knowledge on vector species diversity, availability and types of preferred larval sites can inform comprehensive control strategies such as inclusion of environmental management as a component of integrated vector management. These results have implications for control strategies and suggest a greater need for increased surveillance and research in the region due to ongoing climate change.

## Data Availability

The study is part of a larger project on “Early warning systems for improved human health and resilience to climate-sensitive vector-borne diseases in Kenya”. The data can be requested from the project’s scientific committee through the following e-mail address: climatechange@jooust.ac.ke.

## Abbreviations

ANOVA: analysis of variance
IRS: indoor residual spraying
IVM: integrated vector management
LLINs: long-lasting insecticide-treated nets
LSM: larval source management
RVF: Rift Valley fever
